# Supplementary material for: Network Plasticity as Bayesian Inference
Source: PLoS Comput Biol. 2015 Nov 6;11(11):e1004485. doi: 10.1371/journal.pcbi.1004485 (PMC4636322; doi:10.1371/journal.pcbi.1004485)
Supplement: S1 Text — (PDF) [file pcbi.1004485.s001.pdf]

Supplemental Material to *Network Plasticity as Bayesian Inference*

David Kappel[1], Stefan Habenschuss[1], Robert Legenstein, Wolfgang Maass

[1]these authors contributed equally to this work.

S1 Proof of Theorem 1

We provide here the full proof of Theorem (1) of the main text. For convenience, we first reiterate Eq. (12) and Theorem (1) from the main text. Consider the parameter dynamics (Eq. (12) in the main text)

$$d\theta_i \;=\; \left(b(\theta_i) \frac{\partial}{\partial \theta_i} \log p_{\mathcal{S}}(\boldsymbol{\theta}) + b(\theta_i) \frac{\partial}{\partial \theta_i} \log p_{\mathcal{N}}(\mathbf{x}|\boldsymbol{\theta}) + T\, b'(\theta_i) \right)\, dt \;+ \sqrt{2Tb(\theta_i)}\, d\mathcal{W}_i \tag{S1}$$

(for $i = 1, \ldots, M$). We show that the stochastic dynamics (S1) leaves the distribution

$$p^*(\boldsymbol{\theta}) \equiv \frac{1}{\mathcal{Z}} q^*(\boldsymbol{\theta}) \tag{S2}$$

invariant, where $\mathcal{Z}$ is a normalizing constant $\mathcal{Z} = \int q^*(\boldsymbol{\theta})\, d\boldsymbol{\theta}$ and

$$q^*(\boldsymbol{\theta}) \;=\; p(\boldsymbol{\theta}\,|\,\mathbf{x})^{\frac{1}{T}} \;. \tag{S3}$$

The provided proof applies for standard Wiener processes $\mathcal{W}_i$, where process increments over time $t - s$ are normally distributed with zero mean and variance $t - s$:

$$\mathcal{W}_i^t - \mathcal{W}_i^s \;\sim\; \textsc{Normal}(0, t-s) \;, \tag{S4}$$

where $\mathcal{W}_i^t$ denotes the value of an instantiation of the process at time t.

Theorem 1. *Let $p(\mathbf{x}, \boldsymbol{\theta})$ be a strictly positive, continuous probability distribution over continuous or discrete states $\boldsymbol{x}^n$ and continuous parameters $\boldsymbol{\theta} = (\theta_1, \ldots, \theta_M)$, twice continuously differentiable with respect to $\boldsymbol{\theta}$. Let $b(\theta)$ be a strictly positive, twice continuously differentiable function. Then the set of stochastic differential equations* (S1) *leaves the distribution $p^*(\boldsymbol{\theta})$ invariant. Furthermore, $p^*(\boldsymbol{\theta})$ is the unique stationary distribution of the sampling dynamics.*

Proof. First, note that the first two terms in the drift term of Eq. (S1) can be written as

$$\begin{aligned}
b(\theta_i)\frac{\partial}{\partial\theta_i}\log p_{\mathcal{S}}(\boldsymbol{\theta}) + b(\theta_i)\frac{\partial}{\partial\theta_i}\log p_{\mathcal{N}}(\mathbf{x}|\boldsymbol{\theta}) &= b(\theta_i)\frac{\partial}{\partial\theta_i}\log(p_{\mathcal{S}}(\boldsymbol{\theta})p_{\mathcal{N}}(\mathbf{x}|\boldsymbol{\theta})) && \text{(S5)}\\
&= b(\theta_i)\frac{\partial}{\partial\theta_i}\log p(\mathbf{x},\boldsymbol{\theta}) && \text{(S6)}\\
&= b(\theta_i)\frac{\partial}{\partial\theta_i}\log(p(\theta_i|\mathbf{x},\boldsymbol{\theta}_{\setminus i})p(\mathbf{x},\boldsymbol{\theta}_{\setminus i})) && \text{(S7)}\\
&= b(\theta_i)\left(\frac{\partial}{\partial\theta_i}\log(p(\theta_i|\mathbf{x},\boldsymbol{\theta}_{\setminus i}) + \frac{\partial}{\partial\theta_i}\log p(\mathbf{x},\boldsymbol{\theta}_{\setminus i}))\right) && \text{(S8)}\\
&= b(\theta_i)\frac{\partial}{\partial\theta_i}\log(p(\theta_i|\mathbf{x},\boldsymbol{\theta}_{\setminus i}), && \text{(S9)}
\end{aligned}$$

where $\boldsymbol{\theta}_{\setminus i}$ denotes the vector of parameters excluding parameter θ_i. Hence, the dynamics (S1) can be written as

$$d\theta_i = \left(b(\theta_i)\frac{\partial}{\partial\theta_i}\log p(\theta_i|\mathbf{x},\boldsymbol{\theta}_{\setminus i}) + T\,b'(\theta_i)\right)dt + \sqrt{2Tb(\theta_i)}\,d\mathcal{W}_i \tag{S10}$$

(for $i = 1,\ldots,M$). Eq. (S10) has drift $A_k(\boldsymbol{\theta})$ and diffusion $B_{ik}(\boldsymbol{\theta})$:

$$\begin{aligned}
A_k(\boldsymbol{\theta}) &= b(\theta_i)\frac{\partial}{\partial\theta_i}\log p(\theta_i|\mathbf{x},\boldsymbol{\theta}_{\setminus i}) + T\,b'(\theta_i)\ ,\\
B_{ii}(\boldsymbol{\theta}) &= 2T\,b(\theta_i)\ ,\\
B_{ik}(\boldsymbol{\theta}) &= 0 \quad \text{for } i \neq k\ .
\end{aligned} \tag{S11}$$

Hence, the Itô stochastic differential equations (S10) translate into the following Fokker-Planck equation,

$$\frac{d}{dt}p_{\mathrm{FP}}(\boldsymbol{\theta},t) = \sum_i -\frac{\partial}{\partial\theta_i}\left(\left(b(\theta_i)\frac{\partial}{\partial\theta_i}\log p(\theta_i|\mathbf{x},\boldsymbol{\theta}_{\setminus i}) + T\,b'(\theta_i)\right)p_{\mathrm{FP}}(\boldsymbol{\theta},t)\right) + \frac{\partial^2}{\partial\theta_i^2}\left(T\,b(\theta_i)p_{\mathrm{FP}}(\boldsymbol{\theta},t)\right), \tag{S12}$$

where $p_{\mathrm{FP}}(\boldsymbol{\theta},t)$ denotes the distribution over network parameters at time t. Plugging in the presumed stationary distribution $p^*(\boldsymbol{\theta}) = \frac{1}{\mathcal{Z}}q^*(\boldsymbol{\theta})$ on the right hand side of Eq. (S12), one obtains

$$\begin{aligned}
\frac{d}{dt}p_{\mathrm{FP}}(\boldsymbol{\theta},t) &= \sum_i -\frac{\partial}{\partial\theta_i}\left((b(\theta_i)\frac{\partial}{\partial\theta_i}\log p(\theta_i|\mathbf{x},\boldsymbol{\theta}_{\setminus i}) + T\,b'(\theta_i))\frac{q^*(\boldsymbol{\theta})}{\mathcal{Z}}\right) + \frac{\partial^2}{\partial\theta_i^2}\left(T\,b(\theta_i)\frac{q^*(\boldsymbol{\theta})}{\mathcal{Z}}\right) && \text{(S13)}\\
&= \frac{1}{\mathcal{Z}}\sum_i -\frac{\partial}{\partial\theta_i}\left((b(\theta_i)\frac{\partial}{\partial\theta_i}\log p(\theta_i|\mathbf{x},\boldsymbol{\theta}_{\setminus i}) + T\,b'(\theta_i))\,q^*(\boldsymbol{\theta})\right)\\
&\quad + \frac{\partial}{\partial\theta_i}\left(T\,b'(\theta_i)q^*(\boldsymbol{\theta}) + T\,b(\theta_i)\frac{\partial}{\partial\theta_i}q^*(\boldsymbol{\theta})\right) && \text{(S14)}\\
&= \frac{1}{\mathcal{Z}}\sum_i -\frac{\partial}{\partial\theta_i}\left(b(\theta_i)\frac{\partial}{\partial\theta_i}\log p(\theta_i|\mathbf{x},\boldsymbol{\theta}_{\setminus i})\,q^*(\boldsymbol{\theta})\right) + \frac{\partial}{\partial\theta_i}\left(T\,b(\theta_i)\frac{\partial}{\partial\theta_i}q^*(\boldsymbol{\theta})\right) && \text{(S15)}\\
&= \frac{1}{\mathcal{Z}}\sum_i -\frac{\partial}{\partial\theta_i}\left(b(\theta_i)\frac{\partial}{\partial\theta_i}\log p(\theta_i|\mathbf{x},\boldsymbol{\theta}_{\setminus i})\,q^*(\boldsymbol{\theta})\right) + \frac{\partial}{\partial\theta_i}\left(T\,b(\theta_i)\,q^*(\boldsymbol{\theta})\,\frac{\partial}{\partial\theta_i}\log q^*(\boldsymbol{\theta})\right)\ , && \text{(S16)}
\end{aligned}$$

which by inserting $q^*(\boldsymbol{\theta}) = p(\boldsymbol{\theta}\,|\,\mathbf{x})^{\frac{1}{T}}$ becomes

$$\frac{d}{dt}p_{\mathrm{FP}}(\boldsymbol{\theta},t) = \frac{1}{\mathcal{Z}}\sum_i -\frac{\partial}{\partial\theta_i}\left(b(\theta_i)\,\frac{\partial}{\partial\theta_i}\log p(\theta_i|\mathbf{x},\boldsymbol{\theta}_{\setminus i})\,q^*(\boldsymbol{\theta})\right) + \frac{\partial}{\partial\theta_i}\left(T\,b(\theta_i)\,q^*(\boldsymbol{\theta})\,\frac{1}{T}\frac{\partial}{\partial\theta_i}\log p(\boldsymbol{\theta}|\mathbf{x})\right) \tag{S17}$$

$$= \frac{1}{\mathcal{Z}}\sum_i -\frac{\partial}{\partial\theta_i}\left(b(\theta_i)\,\frac{\partial}{\partial\theta_i}\log p(\theta_i|\mathbf{x},\boldsymbol{\theta}_{\setminus i})\,q^*(\boldsymbol{\theta})\right)$$
$$+ \frac{\partial}{\partial\theta_i}\left(b(\theta_i)\,q^*(\boldsymbol{\theta})\,\frac{\partial}{\partial\theta_i}[\log p(\boldsymbol{\theta}_{\setminus i}|\mathbf{x}) + \log p(\theta_i|\mathbf{x},\boldsymbol{\theta}_{\setminus i})]\right) \tag{S18}$$

$$= \frac{1}{\mathcal{Z}}\sum_i -\frac{\partial}{\partial\theta_i}\left(b(\theta_i)\,\frac{\partial}{\partial\theta_i}\log p(\theta_i|\mathbf{x},\boldsymbol{\theta}_{\setminus i})\,q^*(\boldsymbol{\theta})\right) + \frac{\partial}{\partial\theta_i}\left(b(\theta_i)\,q^*(\boldsymbol{\theta})\,\frac{\partial}{\partial\theta_i}\log p(\theta_i|\mathbf{x},\boldsymbol{\theta}_{\setminus i})\right) \tag{S19}$$

$$= \sum_i 0 = 0\ . \tag{S20}$$

This proves that $p^*(\boldsymbol{\theta})$ is a stationary distribution of the parameter sampling dynamics (S10). Under the assumption that $b(\theta_i)$ is strictly positive, this stationary distribution is also unique. If the matrix of diffusion coefficients is invertible, and the potential conditions are satisfied, the stationary distribution can be obtained (uniquely) by simple integration. Since the matrix of diffusion coefficients is diagonal in our model, the diffusion coefficient matrix is trivially invertible if all diagonal elements, i.e. all $b(\theta_i)$, are positive. Also the potential conditions are fulfilled (by design), as can be verified by substituting (S11) into Equation (5.3.22) in [1],

$$Z_i(\boldsymbol{\theta}) = B_{ii}^{-1}(\boldsymbol{\theta})\left(2A_i(\boldsymbol{\theta}) - \frac{\partial}{\partial\theta_i}B_{ii}(\boldsymbol{\theta})\right) \tag{S21}$$

$$= \frac{1}{2Tb(\theta_i)}\left(2b(\theta_i)\,\frac{\partial}{\partial\theta_i}\log p(\theta_i|\mathbf{x},\boldsymbol{\theta}_{\setminus i}) + 2T\,b'(\theta_i) - 2T\,b'(\theta_i)\right) \tag{S22}$$

$$= \frac{1}{T}\frac{\partial}{\partial\theta_i}\log p(\theta_i|\mathbf{x},\boldsymbol{\theta}_{\setminus i})\ , \tag{S23}$$

and by using that the normalization constant $\mathcal{Z}$ is independent of θ_i we can write

$$Z_i(\boldsymbol{\theta}) = \frac{1}{T}\frac{\partial}{\partial\theta_i}\log p(\theta_i|\mathbf{x},\boldsymbol{\theta}_{\setminus i}) = \frac{1}{T}\frac{\partial}{\partial\theta_i}\left(\log p(\theta_i|\mathbf{x},\boldsymbol{\theta}_{\setminus i}) + \log p(\boldsymbol{\theta}_{\setminus i}|\mathbf{x}) - \log\mathcal{Z}^T\right) \tag{S24}$$

$$= \frac{1}{T}\frac{\partial}{\partial\theta_i}\log\frac{p(\boldsymbol{\theta}|\mathbf{x})}{\mathcal{Z}^T} \tag{S25}$$

$$= \frac{\partial}{\partial\theta_i}\log\frac{p(\boldsymbol{\theta}|\mathbf{x})^{1/T}}{\mathcal{Z}} = \frac{\partial}{\partial\theta_i}\log p^*(\boldsymbol{\theta})\ . \tag{S26}$$

This shows that $\boldsymbol{Z}(\boldsymbol{\theta}) = (Z_1(\boldsymbol{\theta}),\ldots,Z_M(\boldsymbol{\theta}))$ is a gradient. Thus, the potential conditions are met and the stationary distribution is unique. □

For strictly positive $b(\theta)$, the diffusion matrix B (Eq. (S11)) is positive definite. Convergence to the stationary distribution follows then directly for strictly positive $p^*(\boldsymbol{\theta})$ (see Section 3.7.2 in [1]).

References

1. Gardiner CW. Handbook of Stochastic Methods. 3rd ed. Springer; 2004.
