# Supplementary material for: Network Plasticity as Bayesian Inference
Source: PLoS Comput Biol. 2015 Nov 6;11(11):e1004485. doi: 10.1371/journal.pcbi.1004485 (PMC4636322; doi:10.1371/journal.pcbi.1004485)
Supplement: S2 Text — (PDF) [file pcbi.1004485.s002.pdf]

Supplemental Material to *Network Plasticity as Bayesian Inference*

David Kappel[1], Stefan Habenschuss[1], Robert Legenstein, Wolfgang Maass

[1]these authors contributed equally to this work.

S2 Supporting information to Figure 1

For the example likelihood function in Fig. 1A we used a mixture of Gaussian distributions, of the form

$$p_{\mathcal{N}}(\boldsymbol{x}\,|\,\boldsymbol{\theta}) \;=\; p_{\mathcal{N}}(\boldsymbol{x}\,|\,\theta_1)\; p_{\mathcal{N}}(\boldsymbol{x}\,|\,\theta_2)\;, \tag{S27}$$

$$\text{with}\qquad p_{\mathcal{N}}(\boldsymbol{x}\,|\,\theta) \;=\; c\,\textsc{Normal}\left(\theta \;\middle|\; \mu_1, \sigma_1^2\right) \;+\; (1-c)\,\textsc{Normal}\left(\theta \;\middle|\; \mu_2, \sigma_2^2\right)\;, \tag{S28}$$

$$\text{and}\qquad \textsc{Normal}\left(\theta \;\middle|\; \mu, \sigma^2\right) \;\propto\; \exp\left(-\frac{1}{2\,\sigma^2}(\theta-\mu)^2\right)\;, \tag{S29}$$

where $\mu_1 = 0.3$, $\mu_2 = 0.9$, $\sigma_1 = 0.1$, $\sigma_2 = 0.2$ and $c = 0.3$. In Fig. 1D we used a prior $p_{\mathcal{S}}(\boldsymbol{\theta}) = p_{\mathcal{S}}(\theta_1)p_{\mathcal{S}}(\theta_2)$, with $p_{\mathcal{S}}(\theta_i)$ given by a normal distribution ($\mu = 0.3$, $\sigma = 0.35$). A learning rate of $\eta = 0.005$ was used to sampled trajectories which had a length of 50 and 300 time steps in Fig. 1C and F, respectively. In Fig. 1F the time-discrete version of the synaptic sampling algorithm (7) was used, with $N = T = 1$. In Fig. 1C the same dynamics were used, but the diffusion term and the contribution of the prior $\frac{\partial}{\partial\theta_i}\log p_{\mathcal{S}}(\boldsymbol{\theta})$ were set to zero.
