# Supplementary material for: Network Plasticity as Bayesian Inference
Source: PLoS Comput Biol. 2015 Nov 6;11(11):e1004485. doi: 10.1371/journal.pcbi.1004485 (PMC4636322; doi:10.1371/journal.pcbi.1004485)
Supplement: S3 Text — (PDF) [file pcbi.1004485.s003.pdf]

Supplemental Material to *Network Plasticity as Bayesian Inference*

David Kappel[1], Stefan Habenschuss[1], Robert Legenstein, Wolfgang Maass

[1]these authors contributed equally to this work.

S3 Supporting information to *Synaptic sampling improves the generalization capability of a neural network*

S3.1 Restricted Boltzmann machine (RBM)

A RBM consists of two layers of neurons, the visible layer $\boldsymbol{x}$, and the hidden layer $\boldsymbol{z}$. Synaptic connections are formed only between neurons on different layers (Fig. 2A). Weights of synaptic connections are assumed to be symmetric, i.e., the weight value w_{ij} denotes both the weight of the connection from visible neuron x_j to hidden neuron z_i and the weight of the connection from hidden neuron z_i to visible neuron x_j. Neurons in these layers are stochastic non-spiking neurons with binary output. For given outputs of visible neurons $\boldsymbol{x}$, each neuron z_i in the hidden layer computes its output in a stochastic manner

$$z_i = \begin{cases} 1 \quad , & \text{with probability } \sigma(\sum_j w_{ij} x_j + b_i^{\text{hid}}) \\ 0 \quad , & \text{with probability } 1 - \sigma(\sum_j w_{ij} x_j + b_i^{\text{hid}}) \; , \end{cases} \tag{S30}$$

where b_i^{hid} is the bias of hidden neuron z_i. Analogously, for given outputs of hidden neurons $\boldsymbol{z}$, each neuron z_i in the visible layer computes its stochastic output according to

$$x_i = \begin{cases} 1 \quad , & \text{with probability } \sigma(\sum_j w_{ji} z_j + b_i^{\text{vis}}) \\ 0 \quad , & \text{with probability } 1 - \sigma(\sum_j w_{ji} z_j + b_i^{\text{vis}}) \; , \end{cases} \tag{S31}$$

where b_i^{vis} is the bias of visible neuron x_i. Typically, for given outputs of hidden neurons $\boldsymbol{z}$, the output of the whole visible layer is sampled. In total, the parameter vector $\boldsymbol{\theta}$ for the RBM consists of all weight w_{ij} and all biases b_i^{hid}, b_i^{vis}.

S3.2 Evaluation of model log likelihood

The (non-normalized) log likelihood $\hat{\mathcal{L}}(\mathbf{x}\,|\,\boldsymbol{\theta})$ measure in Fig. 2D,F was computed according to the assumed underlying model, given by the Boltzmann distribution. For a dataset $\mathbf{x} = \boldsymbol{x}^1, \ldots, \boldsymbol{x}^N$, we get

$$\hat{\mathcal{L}}(\mathbf{x}\,|\,\boldsymbol{\theta}) \;=\; \log\left(\sum_{n=1}^{N} \sum_{\boldsymbol{z}} \exp\left(\sum_i \sum_j w_{ij}\, x_j^n\, z_i \;+\; b_i^{\text{hid}} z_i \;+\; b_j^{\text{vis}} x_j^n \right)\right) , \tag{S32}$$

where the sum $\sum_{\boldsymbol{z}}$ runs over all possible states of the hidden neurons. This quantity is equivalent to the exact log likelihood up to a normalizing constant $\mathcal{Z}$, i.e. $\hat{\mathcal{L}}(\mathbf{x}\,|\,\boldsymbol{\theta}) = \log p(\mathbf{x}\,|\,\boldsymbol{\theta}) + \mathcal{Z}$. The current sets of network

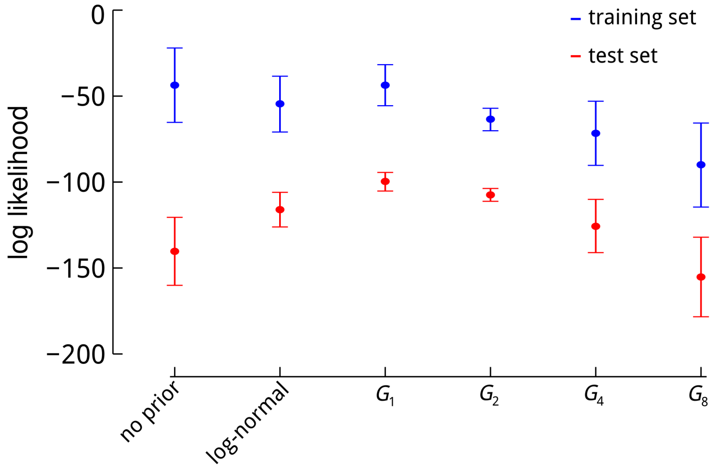

Figure S1: Comparison of the learning performance for different priors. *Learning performances are shown for the training set (blue) and the test set (red). Dots represent average values of the log likelihood after 10000 training steps. The average values were computed based on 100 individual trial runs for each prior distribution. The error bars indicate STD.*

weights and biases were recorded after every 100 update steps and the log likelihood was evaluated for these parameter values. The plots in Fig. 2C-F show linear interpolations between these values. The likelihood plots in Fig. 2D,F show mean and std over 100 individually trained RBMs, all trained and evaluated on the same training and test set.

S3.3 Comparison of the learning performance under different prior distributions

Here we compare the learning performance and generalization capabilities of the Boltzmann machine under different prior distributions. In addition to the uninformative (i.e., uniform) prior on weights (see main text) we used a set of factorized priors for individual weights $p_{\mathcal{S}}(\mathbf{w}) = \prod_{i,j} p_{\mathcal{S}}(w_{ij})$. Here we used a scaled version of the mixture of two Gaussians, given by

$$G_k \;:=\; p_{\mathcal{S}}(w_{ij}) \;=\; 0.5\,\textsc{Normal}\left(w_{ij} \mid k \cdot \mu_1, k \cdot \sigma_1\right) \;+\; 0.5\,\textsc{Normal}\left(w_{ij} \mid k \cdot \mu_2, k \cdot \sigma_2\right)\;, \tag{S33}$$

with means $\mu_1 = 1.0$, $\mu_2 = 0.0$, and standard deviations $\sigma_1 = \sigma_2 = 0.15$. Therefore, G_k denotes a scaled version of the prior used in Fig. 2E, with G_1 being identical to equation (25) of the main text. In addition we used a log-normal prior with location $\mu = 0$ and scale $\sigma = 1.2$ of the form

$$p_{\mathcal{S}}(w_{ij}) \;=\; \frac{1}{w_{ij}\sigma\sqrt{2\pi}} \exp\left(-\frac{1}{2\sigma^2}(\log w_{ij} - \mu + \theta_0)^2\right) \tag{S34}$$

Using these prior distributions we repeated the experiment in Section *Synaptic sampling improves the generalization capability of a neural network* of the main text. A comparison of the performance of the Boltzmann machine for different prior distributions and for the learning scenario without prior is provided in Fig. S1. The choice of the prior can have a significant impact on the learning performance and with respect to overfitting. Nevertheless, we found that most prior distributions had a positive impact on the performance on the test set, thereby decreasing overfitting effects. However, the extreme case of the prior G_8 shows that a bad choice for the prior can result in performance that is worse than without a prior.
