# Supplementary material for: Network Plasticity as Bayesian Inference
Source: PLoS Comput Biol. 2015 Nov 6;11(11):e1004485. doi: 10.1371/journal.pcbi.1004485 (PMC4636322; doi:10.1371/journal.pcbi.1004485)
Supplement: S4 Text — (PDF) [file pcbi.1004485.s004.pdf]

Supplemental Material to *Network Plasticity as Bayesian Inference*

David Kappel[1], Stefan Habenschuss[1], Robert Legenstein, Wolfgang Maass

[1]these authors contributed equally to this work.

S4 Supporting information to *Spine motility as synaptic sampling*

S4.1 Spine motility as synaptic sampling

Here we derive the synaptic sampling model for spine motility given in Eq. (10), of the main text. The theory applies for mapping functions $f : \mathbb{R} \to \mathbb{R}$ which are continuous, strictly monotonic and twice differentiable, such that they uniquely map values from θ_i to w_i, i.e. $w_i = f(\theta)$. One example is the exponential mapping (9) provided in the main text. Further we define $f(\boldsymbol{\theta}) = (f(\theta_1), \ldots, f(\theta_M))$. Let $\boldsymbol{w} = f(\boldsymbol{\theta})$ and thus $p_{\mathcal{N}}(\mathbf{x}|\boldsymbol{w}) = p_{\mathcal{N}}(\mathbf{x}|f(\boldsymbol{\theta}))$. Then the synaptic sampling dynamics, with prior $p_{\mathcal{S}}(\boldsymbol{\theta}) = \prod_i p_{\mathcal{S}}(\theta_i)$ and likelihood $p_{\mathcal{N}}(\mathbf{x}|\boldsymbol{w})$, given by eq. (S1) can be rewritten in the from

$$d\theta_i = \left(b(\theta_i)\, \frac{\partial}{\partial \theta_i} \log p_{\mathcal{S}}(\boldsymbol{\theta}) \;+\; b(\theta_i)\, \frac{\partial}{\partial \theta_i} \log p_{\mathcal{N}}(\mathbf{x}|\boldsymbol{w}) \;+\; T\, b'(\theta_i) \right) dt + \sqrt{2T\, b(\theta_i)}\, d\mathcal{W}_i \tag{S33}$$

$$= \left(b(\theta_i)\, \frac{\partial}{\partial \theta_i} \log p_{\mathcal{S}}(\boldsymbol{\theta}) \;+\; b(\theta_i)\, f'(\theta)\, \frac{\partial}{\partial w_i} \log p_{\mathcal{N}}(\mathbf{x}|\boldsymbol{w}) \;+\; T\, b'(\theta_i) \right) dt + \sqrt{2T\, b(\theta_i)}\, d\mathcal{W}_i \;. \tag{S34}$$

Thus, for the parameter dynamics an additional term $f'(\theta_i) = \frac{\partial}{\partial \theta_i} f(\theta_i)$ has to be taken into account that scales the effect of spike-triggered weight changes.

For the particular choice of the exponential mapping (9) this term evaluates to $f'(\theta_i) = \exp(\theta_i - \theta_0)$. Inserting this and using the simplifying choices of $b(\theta_i) = b$ and $T = 1$, we get

$$d\theta_i = b \left(\frac{\partial}{\partial \theta_i} \log p_{\mathcal{S}}(\boldsymbol{\theta}) \;+\; N \exp(\theta_i - \theta_0)\, \frac{\partial}{\partial w_i} \log p_{\mathcal{N}}(\boldsymbol{x}^n|\boldsymbol{w}) \right) dt \;+\; \sqrt{2b}\, d\mathcal{W}_i \;, \tag{S35}$$

which is the result (10).

S4.2 Resulting log-normal priors over synaptic weights

Throughout all simulations of the spiking WTA circuits we used independent Gaussian priors, $p_{\mathcal{S}}(\boldsymbol{\theta}) = \prod_i \text{NORMAL}(\theta_i \mid \mu, \sigma^2)$ for the synaptic parameters θ_i. We show here that this choice for the prior $p_{\mathcal{S}}(\boldsymbol{\theta})$ together with $b(\theta_i) = b$ and the exponential parameter mapping $w_i = \exp(\theta_i - \theta_0)$ induces a log-normal prior distribution over the synaptic efficacies w_i, parametrized by μ, θ_0 and σ, given by

$$\hat{p}_{\mathcal{S}}(w_i) = \frac{1}{w_i \sigma \sqrt{2\pi}} \exp\left(-\frac{1}{2\sigma^2} (\log w_i - \mu + \theta_0)^2 \right) \;. \tag{S36}$$

First, we derive this result for general mapping functions $f(\cdot)$, which can be formalized in the following

way: For every $f(\boldsymbol{\theta})$, $b(\theta_i)$ and $p_{\mathcal{S}}(\boldsymbol{\theta})$, as defined above, the stochastic dynamics of $\boldsymbol{w} = f(\boldsymbol{\theta})$ can be described explicitly in the sampling space of $\boldsymbol{w}$, and the resulting stochastic differential equations have again the form (S1), with a new set of functions $\hat{b}(w_i)$ and $\hat{p}_{\mathcal{S}}(\boldsymbol{w}) = \prod_i \hat{p}_{\mathcal{S}}(w_i)$, given by (see proof below)

$$
\begin{aligned}
dw_i \;&=\; \left(\hat{b}(w_i)\,\frac{\partial}{\partial w_i}\log \hat{p}_{\mathcal{S}}(\boldsymbol{w}) \;+\; \hat{b}(w_i)\,\frac{\partial}{\partial w_i}\log p_{\mathcal{N}}(\mathbf{x}|\boldsymbol{w}) \;+\; T\,\hat{b}'(w_i)\right) dt \;+\; \sqrt{2T\,\hat{b}(w_i)}\,d\mathcal{W}_i\;, \\
&\text{with} \qquad \hat{p}_{\mathcal{S}}(w_i) \;=\; \frac{p_{\mathcal{S}}\left(f^{-1}(w_i)\right)}{f'\left(f^{-1}(w_i)\right)} = \frac{p_{\mathcal{S}}(\theta_i)}{f'(\theta_i)} \qquad\qquad (S37) \\
&\text{and} \qquad \hat{b}(w_i) \;=\; f'^2\left(f^{-1}(w_i)\right)\, b\left(f^{-1}(w_i)\right) = f'^2(\theta_i)\, b(\theta_i)\;,
\end{aligned}
$$

where $f^{-1}(w_i) = \theta_i$ is the inverse function of $f(\cdot)$. Note that since (S37) is of the form (S1), the proof provided in Theorem 1 for the stationary distribution of $\boldsymbol{\theta}$ applies also to $\boldsymbol{w}$. The unique stationary distribution over the synaptic weights is therefore given by $p^*(\boldsymbol{w}) \equiv \frac{1}{\mathcal{Z}} q^*(\boldsymbol{w})$, with $q^*(\boldsymbol{w}) = \left(\hat{p}_{\mathcal{S}}(\boldsymbol{w})\, p_{\mathcal{N}}(\mathbf{x}|\boldsymbol{w})\right)^{\frac{1}{T}}$.

For the choices $p_{\mathcal{S}}(\theta_i) = \textsc{Normal}(\theta_i \mid \mu, \sigma^2)$, $b(\theta_i) = b$ and $w_i = \exp(\theta_i - \theta_0)$ (thus: $\theta_i = f^{-1}(w_i) = \log w_i + \theta_0$), plugged into the general result (S37), we get

$$
\hat{p}_{\mathcal{S}}(w_i) \;=\; \frac{p_{\mathcal{S}}\left(f^{-1}(w_i)\right)}{f'\left(f^{-1}(w_i)\right)} \;=\; \frac{1}{w_i \sigma \sqrt{2\pi}} \exp\left(-\frac{1}{2\sigma^2}(\log w_i - \mu + \theta_0)^2\right) \qquad (S38)
$$

$$
\text{and} \qquad \hat{b}(w_i) \;=\; f'^2\left(f^{-1}(w_i)\right)\, b\left(f^{-1}(w_i)\right) \;=\; c\,\exp(\log w_i + \theta_0 - \theta_0)^2 \;=\; c\, w_i^2\;. \qquad (S39)
$$

Eq. (S38) is the log-normal distribution and thus recovers the result (S36). Note that (S39) suggests that the resulting diffusion of the synaptic weights grows quadratically with the strength of the synaptic efficacies.

Proof. We prove the result (S37) by deriving the stochastic process that governs $\boldsymbol{w} = f(\boldsymbol{\theta})$. From (S34), we identify the drift $A_i(\boldsymbol{\theta})$ and diffusion $B_{ik}(\boldsymbol{\theta})$ according to

$$
A_i(\boldsymbol{\theta}) = b(\theta_i)\,\frac{\partial}{\partial \theta_i}\log p_{\mathcal{S}}(\boldsymbol{\theta}) \;+\; b(\theta_i)\, f'(\theta_i)\,\frac{\partial}{\partial w_i}\log p_{\mathcal{N}}(\mathbf{x}|\boldsymbol{w}) \;+\; T\, b'(\theta_i) \qquad (S40)
$$

$$
B_{ii}(\boldsymbol{\theta}) = 2T\, b(\theta_i) \qquad \text{and} \qquad B_{ik}(\boldsymbol{\theta}) = 0, \quad \text{for} \quad i \neq k\;. \qquad (S41)
$$

Applying the rule for change of variables for stochastic differential equations to this expression yields (see [1], p. 95f)

$$
\begin{aligned}
dw_i \;&=\; df(\theta_i) = \left(A(\theta_i) f'(\theta_i) \;+\; \frac{1}{2}\, B(\theta_i)\, f''(\theta_i)\right) dt + \sqrt{B(\theta_i)}\, f'(\theta_i)\, d\mathcal{W}_i \\
&=\; \Bigg(f'(\theta_i)\, b(\theta_i)\,\frac{\partial}{\partial \theta_i}\log p_{\mathcal{S}}(\boldsymbol{\theta}) \;+\; f'^2(\theta_i)\, b(\theta_i)\,\frac{\partial}{\partial w_i}\log p_{\mathcal{N}}(\mathbf{x}|\boldsymbol{w}) \;+ \qquad\qquad (S42) \\
&\qquad f'(\theta_i)\, b'(\theta_i) \;+\; f''(\theta_i)\, b(\theta_i)\Bigg) dt + f'(\theta_i)\,\sqrt{2T\, b(\theta_i)}\, d\mathcal{W}_i.
\end{aligned}
$$

By rearranging and expanding the terms we get

$$dw_i = \left(f'^2(\theta_i)\, b(\theta_i) \left(\frac{\frac{\partial}{\partial \theta_i} \log p_{\mathcal{S}}\left(\boldsymbol{\theta}\right)}{f'\left(\theta_i\right)} - \frac{f''\left(\theta_i\right)}{f'^2\left(\theta_i\right)} \right) \; + \; f'^2(\theta_i)\, b(\theta_i) \frac{\partial}{\partial w_i} \log p_{\mathcal{N}}(\mathbf{x}|\boldsymbol{w}) + \right.$$

$$\left. f'\left(\theta_i\right) b'\left(\theta_i\right) \; + \; 2\, f''\left(\theta_i\right) b\left(\theta_i\right) \right) dt + \sqrt{2T\, f'^2(\theta_i)\, b(\theta_i)}\, d\mathcal{W}_i \,.$$

Finally, by using the expressions for $\hat{b}(w)$ and $\hat{p}(w)$, taking the derivatives and comparing the terms we recover the result (S37)

$$dw_i = \left(\hat{b}(w_i) \frac{\partial}{\partial w_i} \log \hat{p}_{\mathcal{S}}(\boldsymbol{w}) \; + \; \hat{b}(w_i) \frac{\partial}{\partial w_i} \log p_{\mathcal{N}}(\mathbf{x}|\boldsymbol{w}) \; + \; \hat{b}'(w_i) \right) dt + \sqrt{2T\, \hat{b}(w_i)}\, d\mathcal{W}_i$$

which completes the proof. □

References

1. Gardiner CW. Handbook of Stochastic Methods. 3rd ed. Springer; 2004.
