# Supplementary material for: Network Plasticity as Bayesian Inference
Source: PLoS Comput Biol. 2015 Nov 6;11(11):e1004485. doi: 10.1371/journal.pcbi.1004485 (PMC4636322; doi:10.1371/journal.pcbi.1004485)
Supplement: S5 Text — (PDF) [file pcbi.1004485.s005.pdf]

Supplemental Material to *Network Plasticity as Bayesian Inference*

David Kappel[1], Stefan Habenschuss[1], Robert Legenstein, Wolfgang Maass

[1]these authors contributed equally to this work.

S5 Supporting information to Figure 3

The survival functions and parameter traces in Fig. 3B,D-F were based on the dynamics of synaptic parameters for prolonged runs of phase 1 of the experiment reported in Fig. 4. The network architecture was as described in Section *Fast adaptation of synaptic connections and weights to a changing input statistics* of the main text. The network inputs were given by different realizations of digit *1* as described in Section *Inherent network compensation capability through synaptic sampling* of the main text. Learning was done for 108 hours of simulated biological time. In Fig. 3F we used 100× slower learning dynamics ($b = 10^{-6}$). Each plot shows the survival of synapses that were newly formed during the preceding 12 hours, i.e. only active synapses that were retraced 12 hours ago are analyzed. The first 48 hours of learning were not evaluated. For the power-law fits, we adapted the method reported in [1]. Power law functions were given by $y = (t+1)^{-\gamma}$, where t is the survival time in hours, y is the fraction of remaining synapses and γ is the decay parameter. To fit γ to the data we measured $t_{1/4}$, i.e. the time it takes until 3/4 of the synapses have decayed. The mean over the three trials shown in Fig. 3E,F was then used to evaluated γ. For the fast dynamics in Fig. 3E this yielded $t_{1/4} = 1.23 \pm 0.58$ hours and $\gamma = 2.00$. For the slow dynamics in Fig. 3F we measured $t_{1/4} = 11.59 \pm 2.54$ hours, resulting in $\gamma = 0.64$.

References

1. Löwenstein Y, Yanover U, Rumpel S. Predicting the dynamics of network connectivity in the neocortex. The Journal of Neuroscience. 2015;35(36):12535–1.
