# Supplementary material for: Network Plasticity as Bayesian Inference
Source: PLoS Comput Biol. 2015 Nov 6;11(11):e1004485. doi: 10.1371/journal.pcbi.1004485 (PMC4636322; doi:10.1371/journal.pcbi.1004485)
Supplement: S6 Text — (PDF) [file pcbi.1004485.s006.pdf]

Supplemental Material to *Network Plasticity as Bayesian Inference*

David Kappel[1], Stefan Habenschuss[1], Robert Legenstein, Wolfgang Maass

[1]these authors contributed equally to this work.

S6 Supporting information to *Inherent network compensation capability through synaptic sampling*

Here we describe the method that was used to evaluate the reconstruction performance, and provide further details to the emergent assembly sequences among the hidden neurons.

S6.1 Evaluating the reconstruction performance

The reconstructed visual stimuli were generated by producing an auditory stimulus via the $\mathbf{x}_A$ neurons and evaluating the corresponding activity of visual $\mathbf{z}_V$ neurons. A sample auditory stimulus from the test set was randomly chosen and spike patterns were generated as for the training session (see main text). The resulting spike trains from the $\mathbf{z}_V$ neurons were smoothed with the EPSP kernel (28). The current strengths of the feedforward synapses were then weighted by these smoothed responses, evaluated 300ms after stimulus onset. Fig. 5B shows two example reconstructed stimuli. The pixel values were rescaled to the color range of the images.

The reconstruction performance was assessed by the performance of linear classifiers trained on the response of $\mathbf{z}_V$ neurons. The classifiers were trained on 50 samples of reconstructed visual stimuli of each digit class, generated by producing each time fresh Poisson trains in the $\mathbf{x}_A$ neurons with time-varying firing rates according to the spoken digit samples, using Matlab's build-in naive Bayes classifier method. Additional 50 samples of each class were then used to evaluate the reconstruction performance (number of correctly labeled samples). The values shown in Fig. 5E are mean values over 20 classifiers, trained and tested for independently generated Poisson spike trains for $\mathbf{x}_A$ neurons as described above.

S6.2 Assembly sequence analysis

To further evaluate the emergent activity patterns and connectivity in the network of hidden neurons we focused on the emergent assembly sequences within the hidden neuron (see e.g. [1] for experimental data on assembly sequences). Affiliation of neurons to assembly sequences was assessed through the PETH (see main text). PETHs were computed for both digits over 100 trial responses from all $\mathbf{z}_A$ and $\mathbf{z}_V$ neurons. Neurons were assigned to the assembly sequence corresponding to the digit for which the neuron showed the maximum PETH amplitude. Neurons for which the maximum was outside the time interval [50ms, 450ms] after stimulus onset, were excluded from the analysis (not assigned to an assembly sequence). We refer to the set of neurons that take part in these assembly sequences in the visual and auditory populations $\mathbf{z}_A$ and $\mathbf{z}_V$ as $V(\mathit{1}), A(\mathit{1})$ for digit *1*, and $V(\mathit{2}), A(\mathit{2})$ for digit *2*. We find that synaptic plasticity generates associations between corresponding

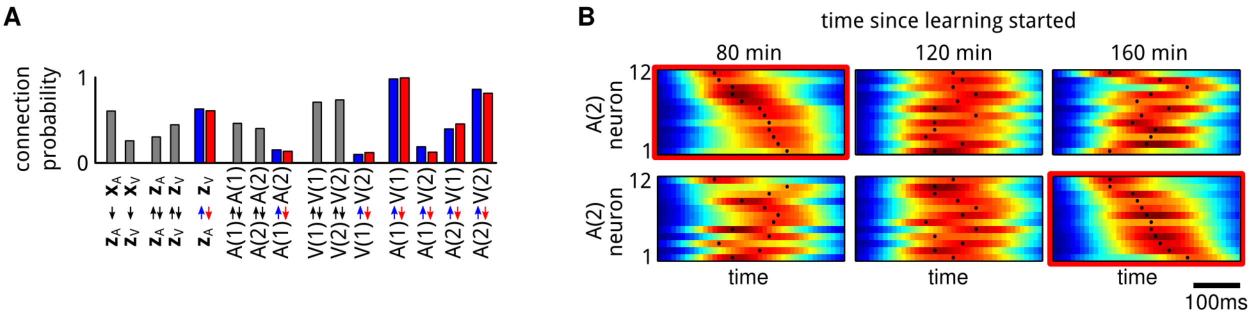

Figure S2: Emergent assembly sequences and functional connectivity in a simplified model of multi-modal sensory integration. *(A) Only a fraction of the structurally possible connectivity (all to all) emerges as functional connectivity after learning in the network shown in Fig. 5 of the main text. Connection probabilities (number of functional connections normalized to the number of possible connections) are shown between input and hidden neurons and between hidden neurons that are recruited for assembly sequences. The colors of the bars match the direction of the connections (colored arrows). Neurons from assembly sequences that encode the same digit class are more likely to be connected after learning. (B) Neurons within the auditory assembly sequence A(2) fire in a characteristic sequential order. PETH evaluated at different training times before the lesions are shown. Neurons are sorted by the time points of highest activity (black dots) after 80 minutes (top) and 160 minutes (bottom) of learning (plot used for sorting is highlighted by red border). The sequential firing order changes during prolonged learning.*

components of the assembly sequences in the visual and auditory ensemble, (i.e., between $V(1)$ and $A(1)$, $V(2)$ and $A(2)$) in spite of the fact that synaptic connections are asymmetric in this model, as in most biological networks of neurons. Fig. S2A shows the connection probabilities between the pairs of assembly sequences after 160 minutes of training. Assemblies that encode correlated stimuli are more likely to be connected.

In Fig. S2B we study the drift of the preferred firing time of neurons within an assembly sequence that takes place on a larger time scale throughout learning, due to the stochastic term in the learning rule. The auditory assembly sequence $A(2)$ is analyzed. Neurons within this assembly sequence fire in a specific order, as in [1]. Our learning model predicts that this sequential order changes during larger periods of learning. The result shown in Fig. S2B is qualitatively similar to the data reported in [2] for a different type of learned neural code (place cells). The time scale of these fluctuations can be regulated through the parameter b (learning rate) in the synaptic sampling rule (3). We had chosen here a faster time scale of hours (rather than days, as in [2]) in order to achieve tractable computer simulation times.

S6.3 Comparison to deterministic STDP

Here we compare the synaptic sampling learning to the approximate spike-based expectation-maximization (EM) algorithm for hidden Markov models implemented through spiking neurons, which was introduced in [3]. The algorithm is a deterministic STDP-like update scheme and realizes the data-dependent drift term of the synaptic sampling rule (11). More precisely we used deterministic updates of the form

$$dw_i \;=\; b\,N\,S(t)\,(x_i(t) - \alpha\,e^{w_i}) \tag{S1}$$

for the lateral and feedforward synaptic weights of the WTA networks.

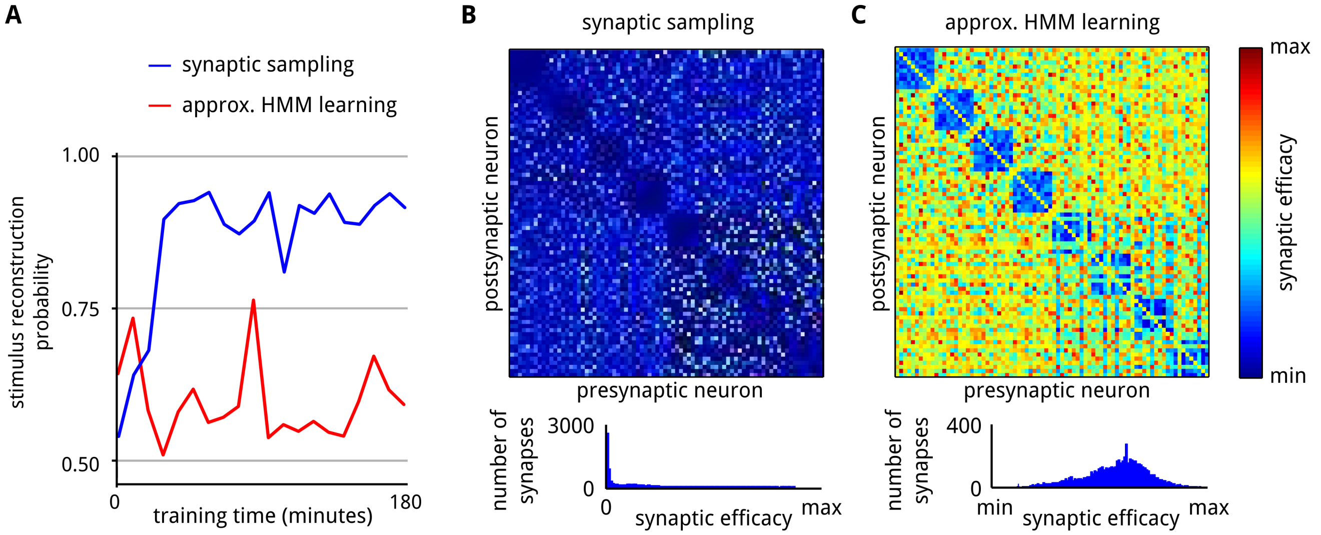

Figure S3: Comparison between synaptic sampling and approximate HMM learning. *(A) The stimulus reconstruction performance of synaptic sampling (blue) and the approximate HMM learning (red). (B,C) Comparison of the lateral synaptic weights that result from synaptic sampling (B) and approximate HMM learning. Insets on the bottom show the histograms over the synaptic parameters.*

We trained a network using the the approximate HMM learning rule (S1) on phase 1 of the learning task in Fig. 5. The results are compared in Fig. S3A. Approximate HMM learning was not able to learn this task accurately which results in a low reconstruction performance throughout the whole learning time. In Fig. S3B,C we compare the matrices of synaptic weights that result from the two algorithms. The color ranges are rescaled to the min/max values of the synaptic weights for each plot. Note that learning rule (S1) – unlike synaptic sampling – can produce negative synaptic weights. Due to the prior distribution over synaptic parameters the representation learned by synaptic sampling is much sparser and associations are more pronounced which allows for a more reliable recall of input stimuli.

S6.4 Impact of the temperature on the reconstruction performance

In Fig. S4 we analyze the impact of the temperature (parameter T in equation (12) of the main text) on the reconstruction performance in the last phase of the learning task in Fig. 5 (all lateral synapses are removed). The speed of the regrowth of retracted synapses is determined by the prior and the random fluctuations due to the Wiener process. Therefore we found that the temperature parameter T has a large impact on the time it takes until the network recovers from the lesion.

With a temperature of zero (deterministic updates) the network requires significantly more time until the reconstruction probability starts to increase. The prior that is close to zero only very slowly drives a sufficient number of synapses above the threshold. With increasing temperature the speed to recover from the lesion increases. For too large temperatures (e.g. $T = 8$) the performance degrades since the network diffuses quickly from solutions. The optimal value for the temperature was found to be between $T = 2$ and $T = 4$ for this particular learning problem (see Fig. S4).

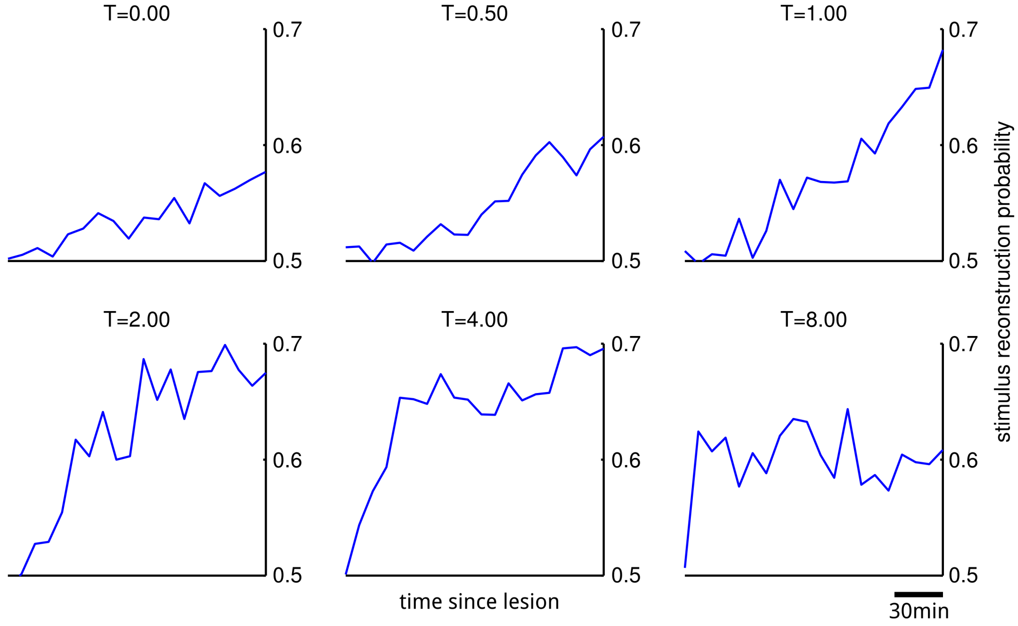

Figure S4: Comparison of the reconstruction performance of networks with different learning temperatures T**.** *The plots show mean values over 20 individual trial runs as the one shown in Fig. 5. Reconstruction performance was evaluated in an 8 minute interval. Plots show linear interpolations between these values.*

References

1. Harvey CD, Coen P, Tank DW. Choice-specific sequencis in parietal cortex during a virtual-navigation decision task. Nature. 2012;484:62–68.

2. Ziv Y, Burns LD, Cocker ED, Hamel EO, Ghosh KK, Kitch L, et al. Long-term dynamics of CA1 hippocampal place codes. Nature Neuroscience. 2013;16(3):264–266.

3. Kappel D, Nessler B, Maass W. STDP Installs in Winner-Take-All Circuits an Online Approximation to Hidden Markov Model Learning. PLoS Computational Biology. 2014;10(3):e1003511.
